# EGFR Signaling in Liver Diseases

**DOI:** 10.3390/ijms17010030

**Published:** 2015-12-29

**Authors:** Karin Komposch, Maria Sibilia

**Affiliations:** Institute of Cancer Research, Department of Medicine I, Comprehensive Cancer Center, Medical University of Vienna, Borschkegasse 8a, A-1090 Vienna, Austria; karin.komposch@meduniwien.ac.at

**Keywords:** EGFR, liver, partial hepatectomy, hepatocellular carcinoma

## Abstract

The epidermal growth factor receptor (EGFR) is a transmembrane receptor tyrosine kinase that is activated by several ligands leading to the activation of diverse signaling pathways controlling mainly proliferation, differentiation, and survival. The EGFR signaling axis has been shown to play a key role during liver regeneration following acute and chronic liver damage, as well as in cirrhosis and hepatocellular carcinoma (HCC) highlighting the importance of the EGFR in the development of liver diseases. Despite the frequent overexpression of EGFR in human HCC, clinical studies with EGFR inhibitors have so far shown only modest results. Interestingly, a recent study has shown that in human HCC and in mouse HCC models the EGFR is upregulated in liver macrophages where it plays a tumor-promoting function. Thus, the role of EGFR in liver diseases appears to be more complex than what anticipated. Further studies are needed to improve the molecular understanding of the cell-specific signaling pathways that control disease development and progression to be able to develop better therapies targeting major components of the EGFR signaling network in selected cell types. In this review, we compiled the current knowledge of EGFR signaling in different models of liver damage and diseases, mainly derived from the analysis of HCC cell lines and genetically engineered mouse models (GEMMs).

## 1. Epidermal Growth Factor Receptor (EGFR) and Its Ligands

The epidermal growth factor receptor (EGFR, also known as ErbB1 or HER-1) is a transmembrane receptor that belongs to the family of receptor tyrosine kinases (RTK) [1]. Structurally, the EGFR is composed of an extracellular domain, where EGFR ligands bind to, followed by a transmembrane domain and an intracellular domain, where the tyrosine kinase domain and the carboxy-terminal tail containing key tyrosine residues are located [2,3]. Ligands that can bind EGFR include epidermal growth factor (EGF), transforming growth factor α (TGF-α), amphiregulin (AR), epiregulin (EREG), betacellulin (BTC), heparin-binding EGF (HB-EGF) and epigen (EPGN) [4]. These ligands contain EGF-like domains that confer binding specificity and additionally contain different motifs, such as sites for heparin binding or glycosylation or an immunoglobulin domain [5]. All ligands are synthesized as transmembrane precursors and are proteolytically cleaved from the surface of the membrane [6] by enzymes that belong to “a disintegrin and metalloproteinases” (ADAM) family, which are also membrane-anchored proteins with metalloprotease activity [7,8,9]. Among the different ADAM family members involved in EGFR ligand cleavage, ADAM17, also known as tumor necrosis factor α (TNF-α)-converting enzyme (TACE), is supposed to play a key role [7,8].

Upon ligand binding, EGFR can form homo- or heterodimers with other EGFR family members. Following activation of the intrinsic kinase domain, several proteins containing Src-homology 2 domains (SH2) such as growth factor receptor-bound protein 2 (Grb2), SHC-transforming protein (SHC), and phospholipase C γ (PLCγ) can bind to the phosphorylated tyrosine residues within the EGFR and activate complex downstream signaling cascades [1,10,11]. The main activated downstream signaling pathways are the Ras-Raf-MEK-ERK1/2 and the signal-transducer and activator of transcription (STAT) 3 and 5 pathways controlling proliferation and differentiation and the phosphatidylinositol-3-kinase (PI3K)-Akt-mechanistic target of rapamycin (mTOR) pathway controlling survival [1,10,11].

The EGFR can form complexes also with other RTKs to initiate signaling, such as platelet-derived growth factor receptor (PDGFR) [12,13], insulin-like growth factor 1 receptor (IGF1-R) [14] or hepatocyte growth factor receptor (c-Met) [15]. This can occur via ligand-dependent or ligand-independent mechanisms, in the latter case physical interaction of receptors with EGFR is believed to be required [12,14]. Moreover, EGFR signaling can be transactivated by several other receptor families, like by cytokine receptors such as interferon-γ-bound receptors and growth hormone-bound receptors [16,17] as well as integrins via Src kinases [18]. G-protein-coupled receptors (GPCRs) have been shown to transactivate EGFR via ADAM protease activation and cleavage of EGFR ligands [7,8,19,20,21]. Furthermore, ligands for GPCRs [20,21], growth hormone (via Src activation) and prolactin (via Janus kinase 1 (Jak1) activation) can phosphorylate EGFR in a ligand-independent manner [22]. In addition, bile acids have been shown to transactivate EGFR [23].

## 2. EGFR and Its Ligands during Liver Development

Several groups generated knockout mice deficient of EGFR and its ligands [24]. AR, BTC, EGF, EREG and EPGN knockout mice did not show any overt phenotype or histological abnormalities [25,26,27,28], beside a mild mammary gland phenotype observed in virgin AR knockout mice [25]. Conversely, more than half of the HB-EGF-deficient mice died before weaning, with survivors showing severe heart abnormalities [27,29]. TGF-α-deficient mice displayed abnormal skin architecture, curly hair and whiskers and an open eye phenotype at birth, whereas several other tissues did not show altered appearance of structure and function [30,31]. AR, EGF and TGF-α triple knockout mice developed similar but more severe abnormalities than TGF-α-deficient mice regarding eye and skin abnormalities. However, triple knockout mice survived for one year showing no additional overt abnormalities [25]. HB-EGF and BTC double knockout mice displayed a more severe heart phenotype compared to HB-EGF single knockout mice [27].

Loss-of-function studies of EGFR point to an indispensable role for EGFR during embryonic development, as EGFR knockout neonates showed severe growth retardation. Depending on the genetic background, EGFR-deficient mice died between mid-gestation and postnatal day 20 after birth [32,33,34]. Concerning liver development, we reported that histological examination of the liver at embryonic day 18.5 and in newborn mutant fetuses revealed no obvious abnormalities [33], whereas Threadgill *et al.*, observed that by postnatal day 8 livers had thickened hepatocyte cords, distorted sinusoidal anatomy, and abnormally vacuolizatized nuclei [34]. Further studies from our laboratory using conditional knockout mice revealed that perinatal deletion of EGFR in hepatocytes alone with the transgenic Alfp-Cre line resulted in reduced body size and weight, which became apparent from the third postnatal week onwards [35]. However, besides growth retardation and body size reduction, mice did not show any liver abnormalities. EGFR deletion in the liver around postnatal day 9 by employing the polyinosinic:polycytidylic acid (polyI:C)-inducible Mx-Cre transgenic line did not lead to growth retardation. However, absence of the EGFR protein in the liver of these mice occurred only 3–4 weeks after induction of recombination, providing a possible explanation for the different phenotype compared to Alfp-Cre-mediated deletion [35]. These results suggest that EGFR and its ligands are not crucial for embryonic liver development.

## 3. EGFR and Its Ligands in Liver Regeneration

Upon tissue damage, the liver can activate an incommensurable repair machinery aimed at protection of liver tissue and restoration of the damaged tissue mass [36]. Profound evidence has accumulated over the years corroborating an indispensable role for EGFR in liver repair and regeneration. Among all tissues, the EGFR is highest expressed in hepatocytes of the adult liver [37], indicating an important role in maintaining liver function. EGF has been shown to be mitogenic in unchallenged livers of rats when infused exogenously [38]. Treatment of cirrhotic rats with EGF and insulin after partial hepatectomy accelerated liver DNA synthesis [39]. EGFR ligands like TGF-α, AR, HB-EGF and EREG induced strong mitogenic signals in cultured hepatocytes [26,40,41,42,43,44,45,46,47,48]. Protein levels of TGF-α, AR, HB-EGF and EREG increased rapidly after partial hepatectomy [26,43,49,50] as well as ADAM17 expression [51]. Furthermore, AR and HB-EGF substantially contributed to liver regeneration after partial hepatectomy as knockout of AR and HB-EGF led to desynchronized S phase entry of hepatocytes [43,52], whereas loss of EREG or TGF-α had no effect [26,53]. However, TGF-α improved hepatic DNA synthesis after hepatectomy in rats with carbon tetrachloride (CCl_4_)-induced cirrhosis [54]. These results suggest that EGFR ligands play different roles during liver regeneration. Furthermore, loss of some EGFR ligands impairs liver regeneration more severely than loss of others, which is probably attributable to redundant expression.

Following partial liver transplantation, whereby the livers of mice were reduced to 50% and transplanted, an increase in AR expression was observed, which resulted in EGFR phosphorylation and induction of proliferation. Injection of AR was able to stimulate liver regeneration even in 30% grafts. Administration of an AR neutralizing antibody attenuated liver regeneration and inhibition of EGFR suppressed liver regeneration in 50% grafts [55]. However, cell transplantation of TGF-α overexpressing hepatocytes could preserve hepatocyte function, as these hepatocytes showed higher proliferation rates [56]. In line with this, overexpression of HB-EGF in transgenic mice led to an increase in proliferating hepatocyte numbers [57]. Interestingly, ablation of salivary glands, which are the main source of EGF in rodents, blocked hepatocytes in the G1 phase. This can be overcome by the administration of EGF [58,59,60]. These results highlight the potential therapeutic use of EGFR ligands to induce liver regeneration.

**Figure 1:**
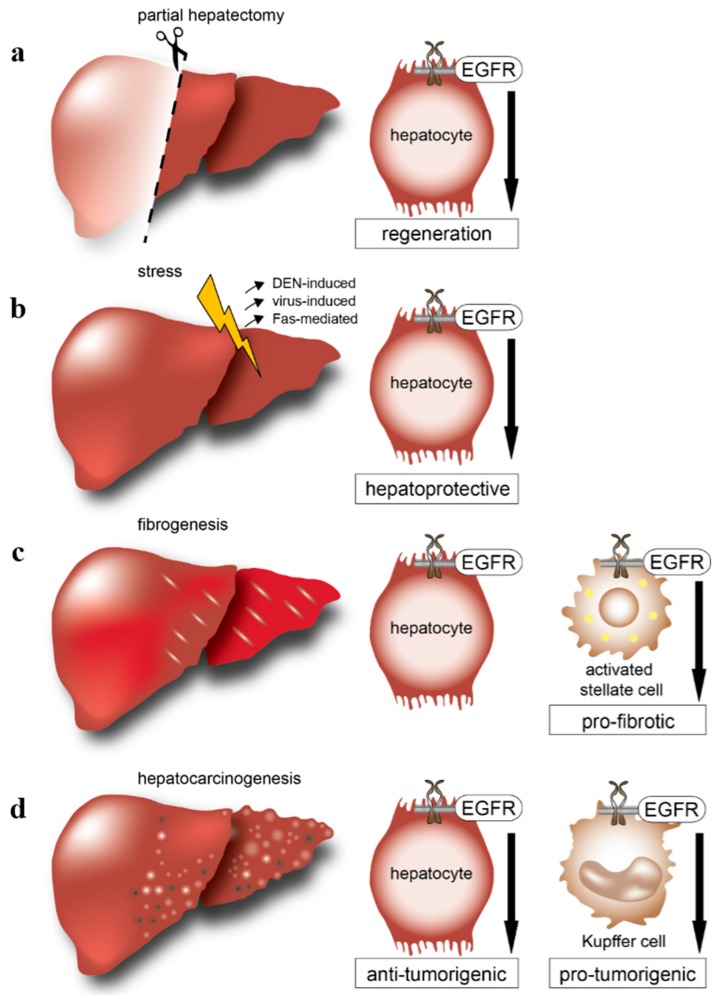
Overview of cell-type-specific roles of epidermal growth factor receptor (EGFR) during liver injury and disease. (**a**) After partial hepatectomy, EGFR is crucial in hepatocytes for liver regeneration; (**b**) During stress, liver injury models induced by diethylnitrosamine (DEN), viruses or Fas, the EGFR plays a hepatoprotective role, but the underlying signaling mechanisms remain unclear; (**c**) After toxic liver injury, the EGFR is dispensable in hepatocytes but essential on activated hepatic stellate cells to induce fibrosis; (**d**) During hepatocarcinogenesis, EGFR is suggested to play an anti-tumorigenic role in hepatocytes, whereas EGFR in Kupffer cells/liver macrophages plays a pro-tumorigenic role.

EGFR binding and EGFR mRNA levels in the liver peak by about threefold 8 hours after partial hepatectomy [61]. The role of EGFR in liver regeneration has been studied in liver- or hepatocyte-specific knockout mouse models [35,62], in transgenic mice expressing dominant negative mutants of EGFR [63], in rats by RNAi injection [64] and in mice using EGFR inhibitors [65]. Inhibition of EGFR with anti-EGFR monoclonal antibodies after partial hepatectomy showed no effect on liver regeneration and cellular proliferation [65]. In EGFR-deficient livers, regeneration was impaired after 2/3 hepatectomy showing reduced cyclin D1 expression and impaired G1-S phase entry, demonstrating that EGFR is a critical regulator of hepatocyte proliferation in the initial phases of liver regeneration [35] (Figure 1a). In contrast to this, it was shown that liver regeneration after 70% hepatectomy in mice that lack EGFR specifically in hepatocytes revealed only a mild phenotype with no change in cyclin D1 expression and only slight differences in cyclin A expression compared to control livers [62]. However, a delay in proliferation could be observed in both studies, but was more pronounced in the first study [35]. In line with this, a recently published study employing a novel transgenic mouse model expressing a dominant negative mutant of EGFR revealed a critical role for the catalytic activity of EGFR during early stages of liver regeneration following partial hepatectomy [63]. Although no mortality and delay of liver regeneration was observed in EGFR-silenced rats after partial hepatectomy, shEGFR treatment suppressed mitosis and proliferation. However, limitations of this system comprise the fact that EGFR messenger RNA (mRNA) and protein were not completely absent [64]. These results consistently demonstrate that EGFR is a critical regulator of hepatocyte proliferation in the initial phases of liver regeneration (Figure 1a).

EGFR can also be indirectly activated during liver regeneration. For example, growth hormone (GH) signaling was shown to control EGFR mRNA expression in the liver from G0 to mid G1 phase transition [66]. Furthermore, GH-receptor-deficient mice showed impaired liver regeneration after partial hepatectomy, which was suggested to be due to impaired EGFR activation [66]. After partial hepatectomy, downregulation of GH and EGFR was observed in two different mouse models of steatosis, a genetic (ob/ob mice) and a methionine- and choline-deficient (MCD) diet model, which could be partially rescued by the administration of GH, which in turn was associated with the restoration of EGFR expression in the liver [67]. Reduced EGFR expression was also responsible for impaired liver regeneration when β-catenin was specifically deleted from hepatocytes [68]. After partial hepatectomy, matrix metallopeptidase 9 (MMP-9) deficiency was reported to impair liver regeneration through inhibition and delay of EGFR activation. Additionally, in MMP-9 knockout mice, the EGFR ligands HB-EGF and AR were expressed at lower levels [69]. Different mechanisms that indirectly lead to EGFR downregulation add to the complexity of this important growth factor signaling pathway, but open the way for new therapeutic treatment options via indirect EGFR modulation.

## 4. EGFR and Its Ligands in Experimental Models of Acute Liver Damage

In a model of Fas-mediated liver injury, AR, EREG and TGF-α were found to be upregulated [70], whereas further administration of AR to mice abrogated Fas-mediated liver injury and showed direct anti-apoptotic effects in primary hepatocytes [70]. AR is undetectable in the healthy liver [43], but is induced by inflammatory signals, where AR was shown to attenuate hepatic acute-phase gene expression [71]. Overexpression of TGF-α has shown a strong protective effect on Fas-mediated hepatic apoptosis [56,72]. In line with this, hepatic *HB-EGF* gene transduction *in vivo* could show therapeutic effects for Fas-induced liver injury [73]. Moreover, loss of ADAM17 in hepatocytes promoted Fas-induced apoptosis, which led to the conclusion that ADAM17 is protective against Fas-mediated liver injury in part also because of increased shedding of EGFR ligands, which can be hepatoprotective. EGFR signaling further protected against Fas-mediated hepatotoxicity [74], whereas cyclooxygenase-2 (COX-2) could prevent Fas-induced hepatocyte apoptosis and liver failure partially through EGFR upregulation [75]. These results demonstrate that the EGFR signaling system is hepatoprotective against Fas-mediated liver injury (Figure 1b).

The EGFR was also shown to be hepatoprotective following treatment with diethylnitrosamine (DEN), a chemical carcinogen commonly used to induce HCC formation in rodents. We reported that mice lacking EGFR in hepatocytes showed increased levels of serum aspartate transaminase (AST) and serum alanine transaminase (ALT), markers for acute liver toxicity [76]. In line with this, damaged areas were significantly increased in livers lacking EGFR in hepatocytes with higher levels of cleaved caspase 3. Stimulation of isolated hepatocytes with DEN resulted in a strong necrotic response [76], which points to a cell-autonomous effect. Furthermore, EGFR-deficient hepatocytes were more sensitive to tumor necrosis factor α (TNF-α) and cycloheximide (CHX) treatment. These results show that EGFR signaling protects from apoptosis and necrosis during DEN-induced liver damage, highlighting the important hepatoprotective role of EGFR [76] (Figure 1b).

## 5. EGFR and Its Ligands in Experimental Models of Chronic Liver Damage

Non-alcoholic steatohepatitis (NASH) can occur due to fat deposition in the liver and is together with alcoholic liver disease (ALD) the most common cause of chronic liver diseases in Western countries [77,78]. Liver fibrosis occurs in most types of chronic liver diseases and is characterized by excessive deposition of extracellular matrix (ECM) proteins [79]. Architectural changes of the liver finally lead to cirrhosis, the formation of pre-neoplastic nodules and the development of hepatocellular carcinoma [80]. Therefore, liver injury and concomitant cirrhosis are strongly linked to hepatocarcinogenesis. As the roles of EGFR ligands and its receptor EGFR in fibrosis and cirrhosis are not completely understood, they have been studied in experimental models of chronic liver injury as well as in the liver of cirrhotic patients.

Upon chronic toxic injury of mouse livers via CCl_4_ or thioacetamide intoxication, the hepatic expression of HB-EGF, TGF-α and AR was increased [49,70,81,82,83], although AR was undetectable in healthy murine livers [43]. In line with this, AR-deficient mice developed less fibrosis. In CCl_4_-induced liver fibrosis, AR contributed to the expression of fibrotic markers and stimulated cell proliferation and survival of fibrogenic cells [84]. AR activated human hepatic stellate cells (HSC) and induced proliferation. Conversely, conditional deletion of HB-EGF in the liver accelerated CCl_4_-induced liver fibrosis [85]. HB-EGF was also expressed in primary cultures of murine HSCs, where HB-EGF inhibited HSC activation [86]. Therefore, HB-EGF is suggested to have a suppressive function in experimental liver fibrosis in mice, in contrast to AR. Conversely, it was shown that HB-EGF promotes HSC proliferation via activation of the EGFR and that HB-EGF is a potential therapeutic target in liver fibrosis [87]. *In vitro*, EGF promoted DNA synthesis in CCl_4_-induced primary rat hepatocytes [88], whereas *in vivo*, administration of EGF before CCl_4_ intoxication protected from injury, as it resulted only in minimal changes and a minor rise in serum transaminase levels [89]. Moreover, EGF and fibroblast growth factor 2 (FGF-2) synergistically downregulated the expression of fibrotic markers in human primary activated HSC [90]. Whereas studies indicate that EGF is anti-fibrogenic in toxic fibrosis, AR is suggested to have a pro-fibrogenic role, which highlights how diverse different EGFR ligands exert their functions by probably activating different EGFR downstream signaling pathways.

*In vivo*, the role of EGFR during chronic liver disease was examined by investigating the effect of the EGFR inhibitor erlotinib on toxic (CCl_4_-induced) fibrosis in mice and on biliary fibrosis (induced via bile duct ligation) and cirrhosis (DEN-induced) in rats [91]. Erlotinib decreased hepatocyte proliferation and liver injury, and prevented the progression of cirrhosis and regressed fibrosis in some animals [91]. Surprisingly, loss of hepatocyte EGFR alone had no effect on the regenerative response after CCl_4_-induced liver injury (Figure 1c) [62]. Interestingly, additional loss of c-Met in hepatocytes resulted in enhanced necrosis and delayed liver regeneration compared to loss of hepatic c-Met alone, suggesting that EGFR and c-Met may partially compensate for the loss of each other [62].

However, EGFR is also expressed on HSCs (Figure 1c) [91,92], which is thought to be the major cell type involved in liver fibrosis. Conditional deletion of EGFR in all liver cells including HSCs or in HSCs alone had no effect on the progression of CCl_4_-induced fibrosis and recovery (Komposch *et al*., unpublished data). However, other studies have shown that the EGFR inhibitor erlotinib prevented disease progression by reducing EGFR phosphorylation in HSCs and by lowering the total number of activated HSCs [91]. In a recent study, activated HSCs were treated with an anti-EGFR single chain fragment variable antibody-TRAIL (tumor necrosis factor-related apoptosis-inducing ligand) (scFv425-sTRAIL) fusion protein, which significantly reduced viability and ECM production in activated HSCs, but did not exert its effect on parenchymal cells. Blockade of activated HSCs with a monoclonal anti-EGFR antibody reduced the effect of the scFv425-sTRAIL fusion protein [93]. Another study reported that bone morphogenetic protein 7 (BMP-7) was capable of inhibiting liver fibrosis by suppressing HSCs activation by a mechanism involving EGFR and TGF-β1 inhibition [94]. Interestingly, bile acids were shown to induce HSC proliferation via activation of EGFR *in vitro* [95]. Mechanistically, in quiescent primary rat HSCs hydrophobic bile acids induce nicotinamide adenine dinucleotide phosphate (NADPH) oxidase-driven reactive oxygen species (ROS) generation and subsequent Yes-mediated EGFR activation, which shifts from a proliferative to an apoptotic signal when c-Jun N-terminal kinase (JNK) is activated at the same time [96]. Furthermore, EGFR transactivation occurred in HSCs and was induced by angiotensin II via activation of ADAM17 [97]. Taken together, these results suggest that eliminating activated HSCs from the liver during fibrosis might be a new therapeutic approach, which needs further proof in animal studies.

The roles of the EGFR ligands HB-EGF and EREG have also been studied in models of biliary fibrosis that include bile duct ligation (BDL) and 3,5-diethoxycarbonyl-1,4-dihydrocollidine (DDC) feeding in mice. Conditional deletion of HB-EGF in the liver enhanced liver fibrosis after BDL in mice [98]. In DDC fed mice, EREG was upregulated, which correlated with data obtained from serum from patients with liver injury. In mice overexpressing EREG, proliferation of hepatocytes was significantly increased and the number of liver progenitor cells increased, suggesting that EREG would be a useful biomarker for liver regeneration [99].

In a mouse model of NASH induced by a MCD diet as well as in patient samples of NASH, AR was found upregulated [100]. Conversely, TGF-α overexpression attenuated NASH after MCD feeding of mice partially because of upregulation of matrix metalloproteinase-1 (MMP-1), which could be blocked by gefitinib in a human HSC line *in vitro* [101]. During alcohol-induced liver damage, TGF-α expression increased, which stimulated collagen synthesis when administered on rat HSCs [102]. Interestingly, administration of EGF protected the liver against alcohol-induced liver damage [103]. These results show that the EGFR system plays an important role during acute and chronic liver diseases, which are the pre-stages on the way to hepatocellular carcinoma development.

## 6. EGFR and Hepatocellular Carcinoma

Hepatocellular carcinoma (HCC) is the second leading cause of cancer related death worldwide, whereas in more developed countries it is the sixth leading cause of cancer death. HCC is less common in women than in men [104]. During 2012, approximately 782,500 new cases of liver cancer and 745,500 deaths from liver cancer occurred worldwide. About 50% of new cases and deaths account for China, which is leading the statistics due to a high prevalence of chronic hepatitis B virus infection [104]. Besides hepatitis B virus (HBV) and hepatitis C virus (HCV) infection, other risk factors for liver cancer in Western countries include obesity, type II diabetes, cirrhosis related to heavy alcohol consumption and non-alcoholic liver disease including NASH [105,106,107,108].

EGFR is overexpressed in human cirrhotic liver tissue and HCCs [109,110]. Overexpression of EGFR occurs in 68% of human HCC correlating with aggressive tumors, metastasis, and poor patient survival [111,112,113]. Overexpression of the EGFR ligands TGF-α, EGF, HB-EGF, AR, and BTC as well as ADAM17 has also been observed in human liver tumor cells and tissues [109,110,114,115,116,117]. Although overexpression of EGFR is present in the majority of HCCs, this increased EGFR expression does not correlate with an increase in *EGFR* gene copy number [118]. Interestingly, a functional polymorphism in the *EGF* gene was associated with increased risk for HCC in patients with liver cirrhosis [119,120]. Intriguingly, as HCC occurs more frequently in man than in women, EGFR overexpression frequency in males could be correlated to a subclass of HCCs with polysomy of chromosome 7 [121].

Higher microvessel density was observed in HCC patient samples with EGFR-positive tumor endothelial cells. EGFR expression in tumor endothelial cells correlated with BTC expression in tumor cells, suggesting paracrine signaling [115]. Another study could show that in HCCs and chronic liver diseases the EGFR was mainly located in the sinusoidal endothelial cells [122]. However, there are no reports that describe EGFR expression in endothelial cells in the healthy liver.

## 7. EGFR and Its Ligands in Hepatitis B Virus-Induced Hepatocellular Carcinoma

There have been many different attempts to model human liver cancer in mice by expressing HBV components. The HBV *x* gene and protein are suggested to be involved in the pathogenesis of HBV-induced hepatocellular carcinoma formation, but the underlying mechanisms are unclear. While the HBV *x* gene is often integrated into cellular DNA during hepatocarcinogenesis, the HBV x protein promotes cell cycle progression, inactivates negative growth regulators and inhibits tumor suppressor genes [123]. In human hepatoma-derived cells, transfection experiments using expression vectors of HBV *x* demonstrated that the *x*-gene product is capable of inducing EGFR overexpression [124]. Furthermore, it was shown that the enhancer-x region contributes to the malignant change of liver cells in HBV carriers through activation of specific genes, such as EGFR [125]. Furthermore, transgenic mice expressing the HBV x protein were shown to be more likely to develop carcinogen-induced HCC [126]. In a more recent study, the HBV-encoded x protein indirectly downregulated EGFR expression in HCC cells via microRNA-7, resulting in decreased growth rate of these cells. Restoration of EGFR expression was able to restore the growth rate [127].

Interestingly, in patient tissue with chronic hepatitis B, TGF-α levels were elevated and even higher than in patient tissue with chronic hepatitis C [114]. During disease, TGF-α overexpression seems to be associated with hepatocyte regeneration of hepatitis B surface antigen (HBsAg)-injured hepatocytes. Continued TGF-α expression might induce dysplasia and finally hepatocarcinogenesis [128]. In a novel HBV mouse model, EGFR was found upregulated on intrahepatic regulatory T (Treg) cells, which rendered them more immunosuppressive and more potent in restraining CD8+ T cell-mediated anti-viral activity, resulting in a higher HBV load in hepatocytes [129]. Furthermore, AR was significantly upregulated in HBV-infected livers. *In vitro*, AR promoted the immunosuppressive activity of EGFR-positive Tregs by inhibiting the production of anti-viral components in CD8+ T cells [129]. Thus, the EGFR system is suggested to contribute to immune tolerance and viral amplification after HBV infection.

## 8. EGFR in Hepatitis C Virus-Induced Hepatocellular Carcinoma

Beside HBV infection, the main risk factors for HCC include HCV infection. Every year, 3–4 million new HCV infections occur, whereas 60%–70% of them develop chronic liver diseases, 5%–20% develop cirrhosis due to chronic infection and 1%–5% will die from complications such as HCC. EGFR is frequently overexpressed in the liver of about 50% of chronic hepatitis C (CHC) patients and often together with TGF-α in cirrhotic CHC compared to non-cirrhotic cases [130]. However, the levels of TGF-α and EGF in chronic viral hepatitis were lower compared to HCC [114,131]. Furthermore, EGFR plasma concentrations were significantly higher in HCC patients in the presence of HCV and HBV infection, suggesting that plasma EGFR could serve as a marker for HCC, in particular when carcinogenesis is affected by virus infection [132]. HCV infection can lead to an increase in AR expression in hepatocytes, and it is believed that AR is responsible for efficient HCV assembly and virion release. Furthermore, AR protected infected cells from HCV-induced cell death and facilitated liver cirrhosis and HCC progression [133].

Studies on transgenic mice showed that the HCV core protein plays a key role in hepatocarcinogenesis [134]. *In vitro*, it promoted proliferation of human hepatoma cells by activation of the MAPK/ERK pathway through upregulation of TGF-α transcription via nuclear factor “kappa-light-chain-enhancer” of activated B-cells (NF-κB) [135]. Interestingly, binding of HCV particles to human hepatocytes induced EGFR activation. HCV entry is suggested to be dependent on co-receptor complex formation between CD81 and claudin-1 on the host cell membrane. *In vitro*, EGFR ligands that enhanced the rate of HCV entry were shown to induce EGFR internalization and co-localization with CD81 but not claudin-1. EGFR kinase inhibitors were shown to be successful inhibitors of HCV infection by preventing endocytosis of EGFR, whereas inhibition of EGFR ligand binding or of EGFR downstream signaling pathways did not affect HCV entry [136,137]. Mechanistically, EGFR was shown to regulate HCV entry by the activation of the EGFR/Shc/Grb2/ Harvey rat sarcoma viral oncogene homolog (HRas) signaling pathway [138]. Furthermore, downstream of EGFR, Ras/MEK/ERK led to the activation of MAPK interacting serine/threonine kinase 1 (MKNK1), which was identified as a host factor in HCV entry [139]. Interestingly, the HCV non-structural protein NS5A attenuated EGFR signaling by alteration of the trafficking profile of EGFR [140] and was able to block EGFR degradation [141]. Noteworthy, also bile acids were demonstrated to play a role in EGFR activation during viral infection. Bile acids promoted HCV replication through activation of EGFR and ERK signaling in infected cells, whereas inhibition of EGFR and ERK could attenuate viral replication [142]. Moreover, IFN-α inducible protein 6 (IFI6) was shown to inhibit HCV entry by impairing EGFR-mediated CD81-claudin-1 interactions [143]. In infected cells, erlotinib synergized with IFN-α to impair HCV replication [144]. These studies highlight the important role of EGFR during HCV entry into the target cell and suggest EGFR inhibitors to successfully constrain viral entry and viral replication.

## 9. EGFR and Its Ligands in Genetically Engineered Mouse Models (GEMMs) of Hepatocellular Carcinoma

So far, it has not been possible to model EGFR-induced liver cancer in mice, as all the attempts to generate GEMMs that overexpress EGFR have been unsuccessful [24], whereas transgenic overexpression has been successful for the EGFR ligands EGF [145,146], TGF-α [147,148], AR [149], BTC [150], HB-EGF [151] and EPGN [152]. Livers of transgenic mice that constitutively overexpress human TGF-α from the mouse metallothionein 1 promoter frequently developed multifocal, well-differentiated HCCs [147,148,153] and treatment of these mice with hepatic carcinogens further accelerated HCC development [153]. Treatment of TGF-α-deficient mice with hepatocarcinogens resulted in the development of multiple, but small pre-neoplastic foci indicating that TGF-α plays an important role in the progression of HCC from small foci to large tumors [53]. In addition, ethanol intake promoted hepatocellular lesions that expressed TGF-α [154]. Interestingly, TGF-α and c-myc double transgenic mice have shown accelerated development of hepatic neoplasia when compared to the expression of one of these transgenes. Treatment of TGF-α and c-myc double transgenic mice with DEN and Phenobarbital further accelerated the development of neoplasia in the liver [155,156]. EGF upregulation is observed in cirrhotic liver disease [157] and has been shown to promote hepatocellular carcinogenesis [158], whereas EGF expression in cirrhotic but non-tumoral tissue correlated with reduced post-surgical survival time [159]. Inflammation led to HB-EGF induction in mesenchymal cells, which stimulated DNA replication in premalignant hepatocytes contributing to HCC development in the early stages [160]. Furthermore, silencing of AR in HCC cells led to reduction of EGFR signaling, inhibition of cell proliferation and increased apoptosis [117]. Interestingly, a new regulatory mechanism for EGFR signaling linking inflammatory and tumor-promoting signals in HCC was proposed, as TNF-α was shown to induce AR shedding and therefore EGFR transactivation in HCC cells [161]. In human HCC cells, fibroblast growth factor 19 (FGF-19) induced AR gene expression via activation of β-catenin. These results introduce a novel crosstalk between the EGFR and FGF system, which was also identified as a driver in hepatocarcinogenesis [162].

EGFR ligands are also able to stimulate the expression of connective tissue growth factor (CTGF) [163]. Overexpression of CTGF correlated with poor prognosis suggesting that downregulation of CTGF by inhibition of transforming growth factor β (TGF-β) may offer clinical benefits [164]. Of note, inhibition of reactive oxygen species (ROS) production attenuated growth of liver tumor cells, which coincided with decreased EGFR phosphorylation and downregulation of EGFR and TGF-α [165]. Adding to complexity, TGF-α, HB-EGF, and AR were shown to be released following EGFR transactivation by GPCRs in a variety of cancer cell lines [19,166,167]. Interestingly, mitogen-inducible gene-6 (*Mig-6*), a multi-adaptor protein and mucin-15 (MUC15), which are expressed in epithelial cells, have been identified as negative regulators of EGFR signaling. Downregulation of Mig-6 and MUC15 frequently occurs in human HCC and correlates with EGFR overexpression [168,169].

**Figure 2:**
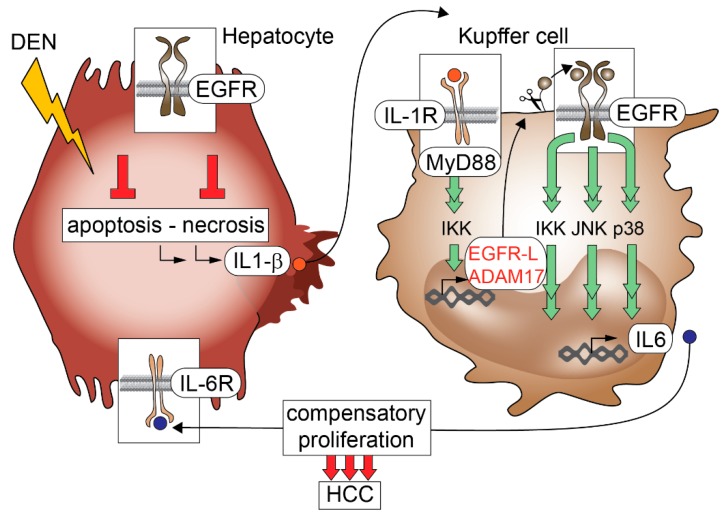
EGFR function in hepatocytes and Kupffer cells during hepatocellular carcinoma (HCC) formation. EGFR signaling is hepatoprotective during diethylnitrosamine (DEN)-induced liver damage. In the absence of EGFR, hepatocytes undergo more necrosis and apoptosis leading to increased IL-1β production, which stimulates Kupffer cells to release IL-6, which is required for compensatory proliferation and repair of damaged hepatocytes. IL-1β-induced IL-6 production in Kupffer cells is dependent on EGFR expression and occurs in a bimodal way involving the activation of the IL-1R/MyD88 pathway to first induce EGFR ligands and ADAM17 expression with subsequent EGFR transactivation required for IL-6 production via c-Jun N-terminal kinase JNK, p38 and inhibitor of κB (IκB) kinase IKK. Red circles: IL-1β, Grey circles: EGFR-ligand, Blue circles: IL-6. Arrows indicate receptor activation.

In a recent study in GEMMs, we identified a pro-tumorigenic role for EGFR in Kupffer cells and/or liver macrophages [76] (Figure 1d). We were able to show that deletion of EGFR in hepatocytes led to increased hepatocarcinogenesis, whereas deletion of EGFR in Kupffer cells/liver macrophages severely reduced the development of HCC in mice (Figure 1d) [76]. Mechanistically, we could demonstrate that damaged EGFR-deficient hepatocytes undergo more necrosis and apoptosis and produce increased levels of IL-1β leading to enhanced stimulation of Kupffer cells, which subsequently produce high levels of IL-6, thus triggering massive compensatory hepatocyte proliferation ultimately leading to increased HCC formation (Figure 2) [76]. These results suggest that EGFR is protective in hepatocytes by suppressing apoptosis and necrosis.

Furthermore, EGFR signaling in hepatocytes prevents excessive IL-1β production and consequent Kupffer cell activation and compensatory proliferation, suggesting an anti-tumorigenic role for EGFR in hepatocytes in this murine model of chemical carcinogenesis [76]. Surprisingly, EGFR-deficient Kupffer cells failed to produce IL-6 in response to IL-1β, therefore leading to reduced HCC formation [76]. In line with this, previous studies have shown that IL-6 is the major driver for hepatocarcinogenesis as IL-6 deletion reduced HCC development [170]. We could furthermore demonstrate that IL-6 production in Kupffer cells following IL-1β stimulation occured in a bimodal way. First IL-1β stimulation of Kupffer cells led to MyD88- and inhibitor of κB (IκB) kinase (IKK)-dependent induction of the EGFR ligands HB-EGF, TGF-α, AR and EREG as well as ADAM17, which is the protease needed for their cleavage. Subsequently, activation of EGFR leads to IL-6 production via JNK, p38 and IKK [76]. Interestingly, the transcriptional regulation of EGFR ligands and ADAM17 following IL-1β stimulation was independent of EGFR signaling [76]. Moreover, EGFR ligands were not induced in Kupffer cells lacking MyD88 indicating that their induction is under direct control of the IL1-receptor signaling pathway [76]. In line with this, after IL-1β stimulation of Kupffer cells *in vitro*, AR was significantly induced in EGFR-proficient and EGFR-deficient Kupffer cells. AR induction was prevented by inhibition of ADAM17 and IKK, but not by inhibition of JNK or p38, indicating that AR release is dependent on IKK-mediated ADAM17 activation (Figure 2) [76]. Furthermore, EGF stimulation of MyD88 knockout Kupffer cells restored IL-6 induction demonstrating that IL-6 production is under direct control of EGFR signaling. Taken together, our data suggest that IL-1β stimulation of Kupffer cells leads to EGFR ligand shedding and subsequent EGFR transactivation, which is required to induce IL-6 release from Kupffer cells [76]. In view of this pivotal role of EGFR in Kupffer cells/liver macrophages during inflammation-driven HCC formation, EGFR-positive Kupffer cells might be a useful prognostic marker and could represent a new therapeutic target for HCC.

## 10. EGFR Inhibitors in Experimental Hepatocellular Carcinoma (HCC)

Inhibiting EGFR either with small molecule inhibitors such as the EGFR tyrosine kinase inhibitors gefitinib or erlotinib or with anti-EGFR antibodies such as cetuximab has given promising results in HCC cell lines and in animal studies. Cetuximab was effective in inhibiting cell cycle and inducing apoptosis in HCC cell lines [171]. In human HCC cells, gefitinib and erlotinib induced growth inhibition, apoptosis and cell cycle arrest [171,172,173]. Erlotinib exerted its effect via MAPK and STAT inhibition, which led to an overexpression of pro-apoptotic and downregulation of anti-apoptotic factors, as well as to a regulation of cell cycle genes towards a G1/G0-arrest [174]. Inhibition of EGFR with gefitinib in rats that developed DEN-induced HCCs following cirrhosis significantly reduced the number of HCC nodules compared to untreated rats [175]. In line with this, erlotinib prevented the progression of cirrhosis in mice and rats and prevented even the development of HCC [91]. In an orthotopic model of HCC, gefitinib inhibited growth of the implanted tumors and intrahepatic metastasis by approximately 50% [176], whereas in another orthotopic model of HCC, gefitinib significantly inhibited the growth of HCC, and the inhibitory effect could be enhanced by combining gefitinib with the cytotoxic agent cisplatin [177]. Gefitinib was able to reduce HCC-induced angiogenesis probably via phosphatase and tensin homolog (PTEN)/Akt signaling [178]. Further studies suggested that gefitinib is able to block the TNF-α-induced transactivation of EGFR via TGF-α shedding by inhibiting MAPKs and Akt [179].

## 11. EGFR Inhibitors in Human HCC

As already mentioned, EGFR overexpression occurs in 68% of human HCCs correlating with aggressive tumors, metastasis, and poor patient survival [111,112,113]. EGFR inhibitors such as gefitinib, erlotinib or lapatinib, which also inhibits ErbB2, have been successful in the treatment of HCC in animal models [91,172,175]. Because of the promising results of EGFR inhibitors in animal models of HCC and their efficacy in other solid human tumors such as non-small cell lung carcinomas and colorectal cancers [180,181,182], it was hypothesized that targeting the EGFR signaling pathway might be beneficial also in HCC. Surprisingly, only modest results could be obtained in clinical studies. Whereas lapatinib and gefitinib treatment were not efficient in the therapy of HCC [183,184,185], erlotinib caused moderate effects when applied as a single agent [184,186,187]. Cetuximab demonstrated no antitumor activity in HCC when applied as a single agent [184,188,189], but elicited some activity in combination with gemcitabine and oxaliplatin in poor prognosis patients [190]. Erlotinib has been tested in combination with the angiogenesis inhibitor bevacizumab in advanced HCC and showed modest activity [191,192] in two studies, but significant and clinically meaningful activity in another trial [193]. Sorafenib, a multikinase inhibitor for vascular endothelial growth factor receptor (VEGFR), PDGFR and Raf) and gefitinib significantly inhibited tumor growth in HCC tumor xenografts in mice [194]. Sorafenib has also shown clinical benefit in advanced HCC. However the SEARCH (Sorafenib and Erlotinib, a Randomized Trial Protocol for the Treatment of Patients With Hepatocellular Carcinoma) trial, the only phase III clinical trial including an EGFR inhibitor, was unable to reveal an improvement in survival of patients with advanced HCC by combining erlotinib and sorafenib [195].

In view of these results, the importance of specifically targeting EGFR signaling in HCCs remains questionable. However, the distinct roles of EGFR in hepatocytes and Kupffer cells during hepatocarcinogenesis discovered by our group possibly shed light on the so far observed treatment failures of HCC with EGFR inhibitors. Indeed, we found that deletion of EGFR in hepatocytes led to increased formation of HCC, whereas deletion of EGFR in Kupffer cells and/or liver macrophages severely reduced development of HCC in mice [76] (Figure 1d). Analysis of two different patient cohorts, one from China with mostly HBV-positive HCCs and one from Europe with mostly alcohol-, HCV- or NASH-induced HCC, which underwent liver transplantation, revealed that EGFR was expressed in liver macrophages of about 45% of HCC patients. Interestingly, EGFR positivity in liver macrophages correlated with poor prognosis with reduced disease free (DFS) and overall survival (OS) of patients [76]. Surprisingly, the expression levels of EGFR in tumor cells did not show any prognostic value for the DFS and OS of patients [76]. We also found that IL-6 levels were increased in the serum of HCC patients of the Asian cohort harboring EGFR-positive Kupffer cells in their tumors and correlating with HBV positivity [76]. Unfortunately, serum IL-6 levels could not be determined in the European cohort. Based on these results, we anticipate that only HCC patients with EGFR-positive macrophages in their tumors would benefit from anti-EGFR therapies, while a worse outcome would be expected in patients where the EGFR is expressed in tumor cells. However, this hypothesis needs to be further validated in clinical trials. Moreover, targeting exclusively EGFR-positive liver macrophages with EGFR inhibitors would be even preferable in HCC patients. Taken together, this study provides a possible explanation for the low success rate of EGFR inhibitors in unstratified patient populations of advanced stage HCC.

Many other possible explanations for treatment failure with EGFR inhibitors are discussed in the literature. One possible explanation for treatment failure could be that mutations of the kinase domain of EGFR do not play a significant role in the tumorigenesis of HCC as activating mutations have not been reported in human HCCs [196,197], whereas for example for non-small cell lung carcinomas the treatment response to gefitinib correlated with an activating mutation in the kinase domain [180]. Interestingly, also epithelial-to-mesenchymal transition (EMT) was examined as a marker for sensitivity or resistance of HCC to EGFR inhibitors. Hepatoma cell lines that were classified as epithelial due to their E-cadherin expression were sensitive to EGFR inhibitors, whereas hepatoma cell lines classified as mesenchymal (vimentin-positive) were resistant to EGFR inhibitors and displayed increased Akt and STAT3 phosphorylation. However, analysis of the EGFR pathway showed that EMT status was independent of EGFR expression or ERK activation [198]. Of note, resistance of human hepatoma cells was also associated with major vault protein (MVP) expression, a protein also known as lung-resistance related protein that is widely distributed in normal tissue and overexpressed in multidrug-resistant cancer cells. MVP decreased sensitivity to the EGFR inhibitor gefitinib via an increase of Akt signaling [199].

Many other growth factors and their receptors are deregulated in human HCC and directly or indirectly influence EGFR signaling. Simultaneous targeting of several growth factor pathways could increase the clinical benefit and reduce development of resistances to targeted therapies. Beside the TGF-α/EGFR signaling pathway, insulin like growth factor-2 (IGF2)/IGF-1R and hepatocyte growth factor (HGF)/c-Met are suggested to play important roles in the development of HCC [108]. Overexpression of IGF-1R was detected in 33% of HCCs and increased activation of IGF-1R was observed in 52% of tumors [200]. The proliferative action of IGF-2 in HCC cell lines required EGFR activation through the release of AR. Inhibition of the IGF-2/IGF-1R signaling pathway potentiated the anti-tumoral effect of gefinitib in HCC. Intriguingly, to overcome IGF-1R inhibition, hepatoma cells were able to activate ErbB3 in an EGFR-dependent manner [201]. These results suggest targeting EGFR and IGF-1R simultaneously in HCC. Furthermore, signaling via HGF/c-Met plays a key role in liver regeneration [202], as HGF is mitogenic for hepatocytes, which is suggested to be a secondary effect to increased processing of EGFR ligands such as TGF-α. Furthermore, EGF and TGF-α were found to stimulate c-Met phosphorylation and nuclear accumulation of β-catenin through EGFR activation in hepatocarcinoma cells [203]. Neutralizing antibodies against TGF-α and/or EGFR could inhibit c-Met phosphorylation. These results suggest that c-Met phosphorylation in tumor cell lines is the result of EGFR activation [15] and promotes parallel inhibition of EGFR and c-Met to increase clinical benefit. In several carcinoma cell lines, but also in an HCC cell lines, HGF was recently found to inhibit EGFR tyrosine kinase activity and that this kinase-inactive EGFR stabilizes cancer-related proteins [204]. Noteworthy, sensitivity to gefitinib could be restored in an EGFR-mutated non-small cell lung carcinoma (NSCLC) cell line by the inhibition of the HGF/c-Met axis [204].

EGFR variant III (EGFRvIII) can be found in human HCC tissue [205], in the serum of HCC patients [206] and in numerous HCC cell lines, where it correlates with rapid cell growth and lower sensitivity to chemotherapeutics like 5-fluorouracil [207]. Treatment of EGFRvIII expressing HCC xenografts with the anti-EGFRvIII monoclonal antibody CH12 inhibited xenograft growth *in vivo* by suppressing tumor proliferation and angiogenesis and by reducing phosphorylation of constitutively active EGFRvIII, Akt, and ERK [208]. Furthermore, CH12 enhanced growth suppression conferred by 5-fluorouracil in HCC xenografts with EGFRvIII expression [209]. Additionally, CH12 was shown to synergize with sorafenib and strongly inhibit the tumor growth of HCC xenografts that expressed EGFRvIII via activation of several growth factor associated downstream signaling pathways [210]. These results suggest targeting EGFRvIII-positive HCCs with CH12 in combination with other targeted drugs as a new promising therapeutic agent.

## 12. EGFR and Its Ligands in Hepatolithiasis and Cholangiocarcinoma

Cholangiocarcinoma (CC) is a primary neoplasm arising from cholangiocytes. Although it is rare, CC is the second most common primary liver neoplasm [211]. Persistent inflammation in the biliary tract strongly predisposes to CC with IL-6 playing a key role during tumorigenesis. IL-6 overexpression increased gene expression and decreased the promoter methylation of EGFR [212]. EGFR overexpression was assessed in different studies with heterogeneous results ranging from 10% to 81% positivity for intrahepatic CC [213,214,215,216,217,218] and approximately 20% for extrahepatic CC [218,219,220]. In a study with 236 cases of CC, EGFR overexpression was associated with lymph node metastasis, tumor stage, lymphatic vessel invasion and perineural invasion in extrahepatic CC. A multivariate analysis revealed EGFR as a significant prognostic marker and a risk factor for tumor recurrence in intrahepatic CC [218]. A recent study showed that EGFR amplification and EGFR overexpression significantly correlated with one another and a multivariate analysis suggested EGFR overexpression being an independent prognostic factor in intrahepatic, but not extrahepatic CC [219]. Concerning the presence of EGFR mutations in CCs, studies have reported opposing results. Whereas one study did not find any somatic mutations of the *EGFR* gene in CC [221], others described somatic mutations in the tyrosine kinase domain of EGFR in a subpopulation of CC [222,223]. EGFR overexpression was also described for hepatolithiasis [224] and associated with the degree of differentiation from hepatolithiasis to intrahepatic CC and with the depth of invasion, which also correlated with poor prognosis [225]. Furthermore, the EMT-like phenotype of cholangiocytes of small bile ducts correlated with strong EGFR expression in the ductular epithelium of hepatolithiasis [226].

In a study with 100 CC patients, cytoplasmic E-cadherin location was associated with EGFR overexpression. In CC cell lines, EGF decreased the expression of E-cadherin and scattering of CC cells, which displayed disrupted adherens junctions. In xenograft tumors, gefitinib treatment restored the membranous expression of E-cadherin [227]. These results suggest that the EGF/EGFR axis triggers EMT in CC cells, highlighting the role of EGFR in CC development. Furthermore, in biliary cancer cells, loss of EBP50, a β-catenin-associating protein, was shown to stimulate EGFR activity to induce EMT-associated features, along with E-cadherin and cytokeratin-19 reduction and induction of the E-cadherin transcriptional repressor Slug as well as loss of cell polarity [228]. EGFR further mediated the phosphorylation of the cytoplasmic tail of the transmembrane mucin MUC1 and this enhanced its affinity for β-catenin potentially correlating with a decrease in cell adhesion and an increase in invasiveness of tumor cells [229,230]. MUC1 was also described to modulate TGF-α-dependent cancer progression and to regulate EGFR stability upon activation and might induce transformation through the inhibition of EGFR degradation [231]. Another study showed that annexin A8, which is highly expressed in CCs but downregulated upon tumor dedifferentiation, is downregulated by activation of the EGF/EGFR system via PI3K and Akt and that this correlated with EMT in CC cells [232].

In a human intrahepatic CC cell line, downregulation of miR-376c, which is suggested to function as a tumor suppressor, accelerated EGF-dependent migration through its direct target growth factor receptor bound protein 2 (GRB2) [233]. Interestingly, in the human intrahepatic CC RBE cell line, EGFR degradation was impaired, which was associated with hypo-phosphorylation of Tyr1045 and with enhanced recycling of EGFR to the cell membrane, indicating that upregulation of Tyr1045 phosphorylation might be a beneficial molecular alteration in EGFR-targeted therapy [234]. A pro-tumorigenic role for the HB-EGF/EGFR axis was recently described in intrahepatic CC. Hepatic myofibroblasts were shown to crosstalk with CC cells and to contribute to tumorigenesis via activation of the HB-EGF/EGFR axis, which could be inhibited by gefitinib [235]. The effect of cetuximab was assessed in two different CC cell lines. Cetuximab did not inhibit cell growth in CC cells carrying a heterozygous Kirsten rat sarcoma viral oncogene homolog (*KRAS*) mutation, but had a dose-dependent effect on growth of CC cells displaying the *KRAS* wild-type [236]. In a rat model of chemical carcinogenesis, EGFR and STAT3 signaling pathways were suggested to contribute to intrahepatic CC. Mechanistically, high STAT3 activity was suggested to be the result of high EGFR activity, which was triggered by TGF-α [237]. *In vitro*, EGFR inhibition or dual inhibition of EGFR and ErbB2 effectively suppressed cell growth and induced apoptosis in human and rodent biliary cancer cell lines and these inhibitors have also been shown to successfully block tumor growth in xenografted athymic nude mice [238]. Given the important role of EGFR in CC development, the employment of EGFR inhibitors was thought to be a promising strategy. However, tyrosine kinase inhibitor therapy showed only modest benefit in certain CC patients [185,239,240,241,242,243,244].

Conjugated bile acids have been described to be increased in CC patient serum [245], which might correlate with biliary tract tumorigenesis [246,247]. In human cholangiocyte cell lines, bile acids were shown to transactivate EGFR in a ligand-dependent manner via a TGF-α, which could be blocked by an MMP inhibitor [23]. Conjugated bile acids in contrast to free bile acids enhanced the activation of NF-κB, which was associated with elevated IL-6 levels and cyclooxygenase (COX-2) expression and inhibition of the turnover of the potent anti-apoptotic protein myeloid cell leukemia 1 (Mcl-1) via an EGFR/Raf1-dependent mechanism [248,249,250,251]. A comparable mechanism has been described in rat HSCs, indicating that bile acids might contribute to enhanced survival and proliferation of myofibroblasts in tumor stroma of CCs [252]. In human CC cells, a crosstalk between COX-2 derived prostaglandin E_2_ (PGE_2_) and EGFR has been demonstrated [253]. EGF stimulation increased cell growth and EGFR kinase inhibitors could successfully decrease COX-2 levels in cultured human CC cells and attenuate cellular growth. This study further demonstrated that CC cells exhibit sustained EGFR activation resulting in extended p42/44 MAPK activation due to defective receptor internalization [254]. These results suggest a positive regulatory loop between EGFR and COX-2. 

## 13. EGFR and Its Ligands in Hepatic Progenitor Cells

During chronic viral hepatitis, alcoholic liver diseases, and non-alcoholic fatty liver disease, hepatic progenitor cells (HPC, also called oval cells) that persist in adulthood, play an important role in the regeneration of hepatocytes and cholangiocytes [255,256,257,258]. Oval cells were described to express EGFR and *in vivo* administration of EGF and HGF enhanced their mitogenic potential after carcinogen administration [259], which highlights the relevance of EGFR in liver progenitor cells. EGFR and c-Met were shown to increase the self-renewal of HPCs through activation of ERK. In adult HPCs, c-Met was a strong inducer of hepatocyte differentiation via Akt and STAT3, whereas EGFR selectively induced NOTCH1 to promote cholangiocyte specification and branching while suppressing hepatocyte commitment. Interestingly, conditional loss of EGFR in the liver rather facilitated than suppressed progenitor-mediated liver regeneration by switching the progenitor cell differentiation to the hepatocyte lineage [260]. EGF administration could significantly increase the proliferation and colony formation of a Sca-1-positive HPC subpopulation, while stimulating the phosphorylation of ERK1/2 and the induction of cyclin D1 [261]. However, while one study described no EGFR expression on carcinogen-treated oval cells of rat liver [262], another study revealed that TGF-α and EGFR were significantly elevated early when oval cells were proliferating. However, expression decreased after a month and remained low until the development of liver tumors [263,264,265]. EGFR-ligand-mediated autocrine mechanisms have been suggested, based on the detection of transcripts of EGFR ligands in oval cells during liver regeneration, but are not yet fully understood [264]. Interestingly, EGF, AR, BTC, HB-EGF and TGF-α were mitogenic for murine oval cell lines, whereas transforming growth factor β1 (TGF-β1), TGF-β2 and TGF-β3 inhibited mitogenesis and induced apoptosis. Combination of EGF ligands and TGF-β factors led to scattering in tissue culture and morphological differentiation in Matrigel [266]. Furthermore, TGF-β1 is an important fibrogenic factor [267] that can induce differentiation of hepatic progenitors into tumor initiating cells via EMT [268,269]. In fetal primary hepatocytes, EGF prevented TGF-β-induced cell death and blocked c-fos induction associated with the apoptotic process induced by TGF-β in these cells [270]. In murine adult hepatic oval cells, EGF suppressed TGF-β-induced apoptosis [271], whereas EGFR inhibition increased TGF-β-induced apoptosis [272], suggesting that constitutively active EGFR might promote proliferation and survival of hepatic progenitor cells. EGF was further shown to suppress and revert TGF-β-1-induced EMT of hepatic progenitors [272]. Taken together, these data highlight the important role of EGFR downstream signaling pathways in regulating the liver progenitor cell compartment.

## 14. Conclusions

Loss of function studies have shown that EGFR and its ligands are not crucial for embryonic liver development. However, EGFR ligands are potent mitogens for cultured hepatocytes. Studies of partial hepatectomy have highlighted the important role of EGFR during liver regeneration and have shown that EGFR is a critical regulator of hepatocyte proliferation in the initial phase of liver regeneration. Interestingly, EGFR ligands have different functions during liver regeneration and loss of some EGFR ligands impairs liver regeneration more severely than loss of others, which is probably attributable to redundant expression. In two different experimental models of acute liver damage, the EGFR signaling system was clearly demonstrated to be hepatoprotective. EGFR ligands exert also different functions during fibrosis, as EGF was shown to be anti-fibrogenic in toxic fibrosis, whereas AR was suggested to have a pro-fibrogenic role. Intriguingly, loss of EGFR alone had no effect on the regenerative response after CCl_4_-induced toxic liver fibrosis. In addition, in other experimental models of liver injury, like biliary fibrosis or NASH, deletion of different EGFR ligands gave opposing results. This further highlights how diverse different EGFR ligands exert their functions by probably activating different EGFR downstream signaling pathways.

However, these results show that the EGFR system plays an important role during acute and chronic liver diseases, which are the pre-stages on the way to hepatocellular carcinoma development. HBV and HCV infection are important risk factors for liver cancer. The HBV *x* gene and protein were suggested to be involved in the pathogenesis of HBV-induced hepatocellular carcinoma formation. While the *x*-gene product was demonstrated to be capable of inducing EGFR overexpression, the HBV-encoded x protein indirectly downregulated EGFR expression in HCC cells. Moreover, the EGFR system was suggested to contribute to immune tolerance and viral amplification after HBV infection. Beside hepatitis B virus infection, the main risk factors for HCC include hepatitis C virus infection. Studies also highlighted an important role of EGFR during HCV entry into the target cell and suggested EGFR inhibitors to be successful inhibitors of viral entry and viral replication. For AR it was shown that HCV infection can lead to an increase in its expression in hepatocytes, and it is believed that AR is responsible for efficient HCV assembly and virion release. Furthermore, AR protected infected cells from HCV-induced cell death and facilitated liver cirrhosis and HCC progression, which indicates a pro-tumorigenic role for AR in HCV-induced HCC.

Besides HBV and HCV infection, other risk factors for liver cancer in Western countries include obesity, type II diabetes, cirrhosis related to heavy alcohol consumption and non-alcoholic liver disease including NASH. In human HCCs, overexpression of EGFR occurs in 68% of HCCs correlating with aggressive tumors, metastasis, and poor patient survival and EGFR ligands were frequently found overexpressed. Inhibiting EGFR either with small molecule inhibitors such as the EGFR tyrosine kinase inhibitors gefitinib or erlotinib or with anti-EGFR antibodies such as cetuximab has given promising results in HCC cell lines and in animal studies. Surprisingly, only modest results could be obtained with EGFR inhibitors in clinical studies. A major drawback in the conductance of therapeutic studies for HCC has been the lack of patient stratification according to biomarkers usually requiring tissue biopsy, which is no longer performed in most centers since the diagnosis is based on radiological imaging criteria. For example, a recent study with the c-Met inhibitor tivantinib nearly failed over significance in preventing tumor progression, since overall only a very moderate effect could be seen. However, closer patient stratification revealed that c-Met high expressing patients showed a survival benefit, while c-Met low patients did not respond to the treatment of tivantinib [273]. A similar patient stratification has not yet been performed in human HCC trials using gefitinib or erlotinib.

The role of EGFR in HCC seems to be more complex than initially believed, particularly because EGFR expression is not restricted to tumor cells, but EGFR was also demonstrated to be expressed on sinusoidal endothelial cells, as well as on Kupffer cells and liver macrophages. Expression of EGFR on liver macrophages correlated with poor prognosis in patient survival. In view of this pivotal role of EGFR during inflammation-driven HCC formation, EGFR-positive Kupffer cells might be a useful prognostic marker and could represent a new therapeutic target for HCC. Since our findings demonstrate that genetic deletion of EGFR in macrophages is sufficient to inhibit HCC development, future studies should consider targeting anti-EGFR therapies specifically to macrophages. Clinical follow-up studies will have to reevaluate the use of EGFR inhibitors in HCC and restrict it to patients with EGFR expression in liver macrophages, since we would expect a worse outcome in patients where the EGFR is expressed in tumor cells. Many other growth factors and their receptors are deregulated in human HCC and directly or indirectly influence EGFR signaling. Simultaneous targeting of several growth factor pathways could increase the clinical benefit and reduce development of resistances to targeted therapies. Since in tumor cell lines, c-Met phosphorylation can result from EGFR activation [15], parallel inhibition of EGFR and c-Met could increase the clinical benefit due to synergistic effects of tivatinib and erlotinib or gefitinib. Since the anti-EGFRvIII monoclonal antibody CH12 was shown to synergize with sorafenib and strongly inhibit the tumor growth of HCC xenografts [210], a possible synergistic effect with erlotinib or gefitinib would need to be evaluated in patients with EGFRvIII expression in HCC biopsy or patient serum. 

EGFR overexpression was also found in CC and was associated with lymph node metastasis, tumor stage, lymphatic vessel and perineural invasion and revealed EGFR as an independent significant prognostic marker and a risk factor for tumor recurrence in CC. Studies suggest that the EGF/EGFR axis triggers EMT in CC cells. Furthermore, a pro-tumorigenic role for the HB-EGF/EGFR axis was described and indirect modulation of TGF-α was associated with CC progression. Despite the apparent important role of EGFR in CC development, EGFR inhibitor therapy revealed also only modest benefit in CC patients and it remains to be investigated whether, similar to HCCs, the EGFR plays a tumorigenic function in non-tumor cells. 

Taken together, there is definitive need for better biomarkers to classify patients into subpopulations that could benefit from anti-EGFR targeted therapies and to closely monitor patient responses. Further work is needed to molecularly dissect different types of liver cancer and to find effective therapies against this deadly disease.

## Abbreviations

ADAM: a disintegrin and metalloproteinases; ALD: alcoholic liver disease; ALT: alanine transaminase; AR: amphiregulin; AST: aspartate transaminase; BDL: bile duct ligation; BMP-7: bone morphogenetic protein 7; BTC: betacellulin; CC: cholangiocarcinoma; CCl_4_: carbon tetrachloride; CHC: chronic hepatitis C; CHX: cycloheximide; c-Met: hepatocyte growth factor receptor; COX-2: cyclooxygenase; CTGF: connective tissue growth factor; DDC: 3,5-Diethoxycarbonyl-1,4-dihydrocollidine; DEN: Diethylnitrosamine; DFS: disease free survival; ECM: extracellular matrix; EGF: epidermal growth factor; EGFR: epidermal growth factor receptor; EMT: epithelial-to-mesenchymal transition; EPGN: epigen; EREG: epiregulin; FGF-19: fibroblast growth factor 19; GEMMs: genetically engineered mouse models; GH: growth hormone; GPCRs: G-protein-coupled receptors; Grb2: growth factor receptor-bound protein 2; HB-EGF: heparin-binding EGF; HBsAg: hepatitis B surface antigen; HBV: hepatitis B virus; HCC: hepatocellular carcinoma; HCV: hepatitis C virus; HRas: Harvey rat sarcoma viral oncogene homolog; IGF1-R: insulin-like growth factor 1 receptor; IKK: inhibitor of κB (IκB) kinase; JNK: c-Jun N-terminal kinase; KRAS: Kirsten rat sarcoma viral oncogene homolog; MCD: methionine- and choline-deficient; Mcl-1: myeloid cell leukemia 1; Mig-6: mitogen-inducible gene-6; MMP: matrix metallopeptidase; mRNA: messenger RNA; mTOR: mechanistic target of rapamycin; MUC: mucin; MVP: major vault protein; NAPDH: nicotinamide adenine dinucleotide phosphate-oxidase; NF-κB: nuclear factor ‘kappa-light-chain-enhancer’ of activated B-cells; NASH: non-alcoholic steatohepatitis; NSCLC: non-small cell lung carcinoma; OS: overall survival; PDGFR: platelet-derived growth factor receptor; PI3K: phosphatidylinositol-3-kinase; PLCγ: phospholipase C γ; polyI:C: polyinosinic:polycytidylic acid; PTEN: phosphatase and tensin homolog; RTK: receptor tyrosine kinases; SH2: Src-homology 2 domains; SHC: SHC-transforming protein; STAT: signal-transducer and activator of transcription; TACE: TNF-α-converting enzyme; TGF: transforming growth factor; TNF-α: tumor necrosis factor α; Treg: regulatory T cells; VEGFR: vascular endothelial growth factor receptor.
